# Promoting global collaboration to improve bioaerosol exposure assessment and understanding of associated health impacts: outcomes from a series of workshops

**DOI:** 10.1099/mic.0.001561

**Published:** 2025-05-15

**Authors:** Emma L. Marczylo, Simon Jackson, Christine Bell, Daniel Andrews, Martin J. D. Clift, Ian Crawford, Gyorgy Fejer, Robert M.W. Ferguson, Matthew C. Fisher, Emma-Jane Goode, James Isaac, Rob Kinnersley, Julie A. Morrissey, Sofya Pozdniakova, Carla Viegas, Andrew Ward, Inge M. Wouters, Frederic Coulon, Zaheer A. Nasir, Philippa Douglas

**Affiliations:** 1 Radiation, Chemical and Environmental Hazards Directorate, UK Health Security Agency, Harwell Campus, Chilton, Oxfordshire, OX11 0RQ, UK; 2 Centre for Environmental Health and Sustainability, University of Leicester, University Road, Leicester, LE1 7RH, UK; 3 School of Public Health, Imperial College London, Michael Uren Building Engineering Hub, White City Campus, Wood Lane, London, W12 0BZ, UK; 4 School of Biomedical Science, Faculty of Health, University of Plymouth, Drake Circus, Plymouth, PL4 8AA, UK; 5 Centre for Facilitation, Liversedge, Yorkshire, WF15 8AZ, UK; 6 In Vitro Toxicology Group, Swansea University Medical School, Faculty of Medicine, Health and Life Sciences, Singleton Park Campus, Swansea University, Swansea, Wales, SA2 8PP, UK; 7 Department of Earth and Environmental Sciences, The University of Manchester, Manchester, M13 9PL, UK; 8 School of Life Sciences, University of Essex, Colchester, CO4 3SQ, UK; 9 MRC Centre for Global Infectious Disease Analysis, Imperial College School of Public Health, Imperial College London, W12 0BZ, London, UK; 10 Chief Scientist’s Group, Environmental Agency, Horizon House, Deanery Rd, Bristol, BS1 5AH, UK; 11 Department of Genetics and Genome Biology, University of Leicester, University Road, Leicester, LE1 7RH, UK; 12 AIRLAB, ISGlobal, Barcelona Institute for Global Health, Barcelona, Spain; 13 Health & Technology Research Center, ESTeSL – Escola Superior de Tecnologia e Saúde, Instituto Politécnico de Lisboa, Lisbon, Portugal; 14 National School of Public Health, Public Health Research Centre, Comprehensive Health Research Center, CHRC, NOVA University Lisbon, Lisbon, Portugal; 15 Central Laser Facility, Science and Technology Facilities Council, Rutherford Appleton Laboratory, Harwell Campus, Didcot, OX11 0QX, UK; 16 Department Population Health Sciences, Institute for Risk Assessment Sciences, Utrecht University, Utrecht, Netherlands; 17 Cranfield University, Faculty of Engineering and Applied Sciences, Cranfield, MK43 0AL, UK

**Keywords:** bioaerosols, BioPM, collaboration, network, workshop

## Abstract

We are surrounded, in both indoor and outdoor environments, by air containing particles of biological origin (bioaerosols). We constantly inhale them, and, depending upon their size, they deposit in different parts of our airways. Despite their ubiquitous nature and our constant exposure, bioaerosol diversity and composition of the environment are not well characterized, and we understand little about which bioaerosols we are exposed to and how this impacts our health, either positively or negatively. Indoor/Outdoor Bioaerosols Interface and Relationships Network (BioAirNet), a Clean Air Programme-funded network, has recognized the need for the bioaerosol community to reflect on the current challenges facing bioaerosol exposure assessment and the determination of the associated cellular/molecular responses driving specific health outcomes. A series of online workshops for the bioaerosol community were hosted by BioAirNet in September 2022, which aimed to bring together global expertise to discuss the current challenges impeding improved assessment of bioaerosol exposure and understanding of the downstream cellular and molecular mechanisms driving health outcomes by discussing these challenges; considering where we need to be, where we are now and how we get there. Professional facilitation was key to their success, enabling the multidisciplinary bioaerosol community to explore and address these challenges within a focused and productive environment to prioritize themes and agree on action plans for continued momentum following the workshops. These themes were as follows: (1) conceptual model; (2) stakeholder mapping; (3) knowledge transfer; (4) writing project and (5) conference-type event, collectively covering research, knowledge mobilization and networking activities. A subsequent in-person follow-up workshop was held in November 2023. It provided an opportunity to share progress on the five themes, critique what had already been done and act as a launch-pad to progress the actions further. Delegates also had the opportunity to share ongoing or upcoming work, particularly projects requiring input from others, to encourage collaborative working and sharing expertise. The use of facilitated workshops is a valuable tool for all scientific communities to collectively explore and successfully address key issues within their field.

## Data availability

Data supporting this study are included within the article and/or supporting materials.

## Introduction

### Bioaerosols

**Fig. 1.**
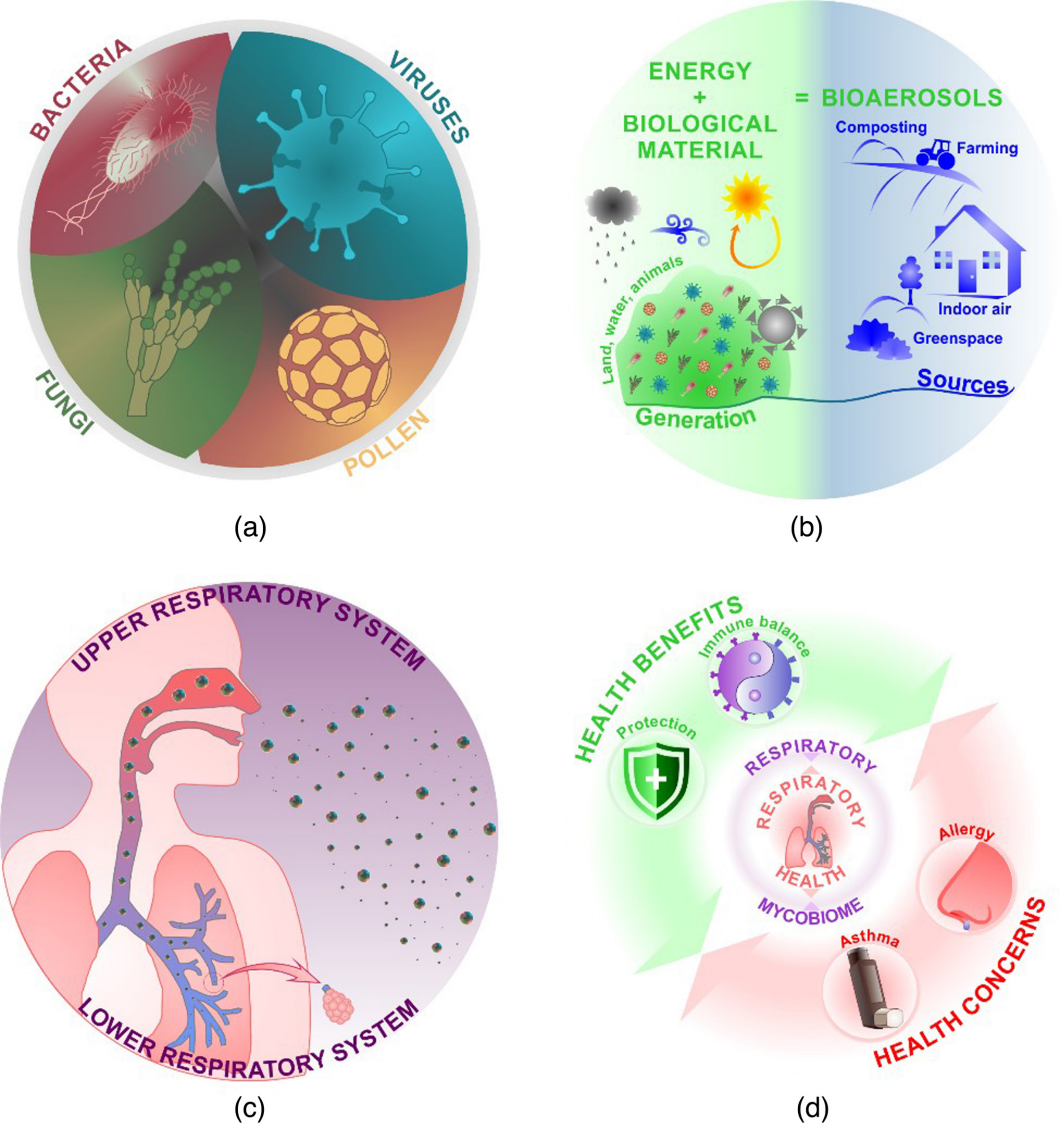
The composition (**a**), generation and sources (**b**), inhalation (**c**) and potential health impacts (**d**) of bioaerosols. Panels (a)–(c) are taken and/or adapted from [21].

Bioaerosols, which are sometimes referred to as biological particulate matter (BioPM), are airborne particles of biological origin, including pollen, bacteria, fungi and viruses and their associated components and products (Fig. 1a) [1]. They are ubiquitous in both indoor and outdoor environments and produced when smaller particles separate from larger sources (e.g. soil, water, trees, plants, people and animals) by natural processes (e.g. meteorological factors and ventilation) or human activities (e.g. composting and waste processing, intensive farming and building occupancy) (Fig. 1b) [2,5]. Bioaerosol particles from environmental sources range in size from <1 to 100 µm and are primarily inhaled. Like all particles, their size determines where in the respiratory system they deposit (Fig. 1c). Respirable particles (<4 µm) can deposit in the small airways (bronchioles) and penetrate into the air sacs within the lungs (alveoli) [6,7].

Unlike air pollutants, environmental bioaerosols can have both positive and negative health outcomes (Fig. 1d). Exposure to a wide diversity of microbes is essential for normal immune system development, helping to reduce the risk of allergy and related conditions such as asthma in later life [8,10]. Inhaled microbes may also colonize the airways, forming a respiratory microbiome and may protect against colonization by pathogens [11]. Exposure to bioaerosols can facilitate or cause infection, inflammation and/or allergy in susceptible individuals or be toxigenic, e.g. produce mycotoxins, some of which are carcinogenic [12]. Which microbes, and in what quantity, facilitate positive vs negative health impacts is poorly understood. Moreover, the role of bioaerosols in the transmission, development and/or exacerbation of disease, the age-dependent context of exposure to bioaerosols and the interaction with other non-biological pollutants is highly complex and requires more detailed research if the health implications of bioaerosol exposure are to be elucidated.

While outdoor pollen is routinely monitored and forecasts are provided to the public, the diversity and composition of other bioaerosols within different outdoor and indoor environments are not as thoroughly characterized. Consequently, our understanding of real-world human exposures is lacking, particularly when considering the complexity of human activities and behaviours that govern time spent by individuals and populations within different environments. This leads to significant variation in exposure, which makes the generation of high-quality and relevant exposure data (both physical measurements and modelling) for human epidemiological studies challenging. In addition, the underlying biological mechanisms linking human exposures with specific health outcomes are also not well understood, highlighting the crucial need for the development and implementation of *ex vivo*/advanced *in vitro* human-relevant models (e.g. air–liquid-interface airway models, lung-on-a-chip or 3D airway models combining both epithelia and immune cells) to comprehensively investigate these complex interactions. Based on the current status of knowledge and understanding in the field, it is imperative that the bioaerosol community reflect upon key knowledge gaps and agree upon steps forward to focus collaborative efforts on applied and impactful solutions to the problems already posed. The focus of these workshops was on bioaerosols from an environmental origin, not particles from a respiratory origin (e.g. those that can transmit contagious diseases such as COVID-19).

### BioAirNet

The Indoor/Outdoor Bioaerosols Interface and Relationships Network (BioAirNet) is one of the six networks funded through the UK Research and Innovation (UKRI) Strategic Priorities Fund (SPF) Clean Air Programme [13]. BioAirNet aims to act as the leading voice for the UK bioaerosols science community by taking a transdisciplinary approach to understanding the complexity and connectivity among people, bioaerosol exposure and resultant health impacts within the indoor–outdoor continuum. The network is split into four themes as follows:

- Theme 1 BioPM sources and dynamics in indoor/outdoor environments.
- Theme 2 BioPM sampling and characterization.
- Theme 3 Human health, behaviour and wellbeing.
- Theme 4 Policy and public engagement.

As such, this network (under Theme 3) was perfectly placed to host a workshop on bioaerosol exposure assessment and the associated cellular/molecular responses and health impacts, and its members were well suited to addressing these challenges.

### Overall aim

The main aim of this series of virtual workshops was to bring together global expertise to discuss the current challenges impeding improved assessment of environmental bioaerosol exposure and understanding of the downstream cellular and molecular mechanisms driving health outcomes, by considering where we need to be, where we are now and how we would get there. It was hoped that the use of professional facilitators would improve focus and productivity, generating achievable action plans and stimulating collaborative work to maintain momentum following the workshops.

## Structure of the workshops

Three interactive, collaborative workshops were coordinated by the Centre for Facilitation [14] and held virtually over three sessions on 14, 22 and 29 September 2022.The team at the Centre for Facilitation specialize in co-design and use a wide range of tools to create a tailored process to facilitate the desired outputs for an event. Each workshop was preceded by a planning and co-design meeting, using the tool SessionLab [15] to enable a transparent approach to co-design. These meetings were attended by the facilitators, the BioAirNet Theme 3 Leads and the BioAirNet Principal Investigator and Network Manager (now collectively referred to as the ‘core team’). A post-workshop review meeting was held on 7 October 2022 and attended by the core team to reflect on the outcomes of the workshops, how these aligned with our initial aims and how to ensure momentum. Our methods were informed and adapted from a previous bioaerosols-related workshop, also facilitated by the team at the Centre for Facilitation [16], and techniques developed from Liberating Structures [17] and Open Space Technology [18].

Members of BioAirNet were invited to the workshop, and the invitation was shared across our own individual networks, including delegates from across the UK and the international community (Canada, The Netherlands, Greece, Switzerland, Spain and Portugal). The workshops were attended by 32 delegates, representing different sectors including academia, government, regulation and industry, covering a wide range of expertise including epidemiology, toxicology, atmospheric physics, exposure assessment, bioaerosol monitoring and measurement, climate change, molecular and microbiology, environmental and public health, chemistry, metagenomics and air quality.

The online workshops were held on Zoom [19], and the digital whiteboard, Mural [20], was used by delegates and facilitators to capture the discussion and enable innovative and creative thinking in a collaborative way, both during and after the workshops (it was recognized that some delegates may have some additional views they wished to share following reflection of the workshops, and that not all delegates could attend every workshop, but could still contribute in their own time). Reflective pauses, breaks and opportunities for wider discussion were built into the workshop agendas, to allow flexibility and freedom for delegates to communicate their views. As the collaborative nature of the workshops depended on all delegates being comfortable using the digital tools, a pre-workshop briefing session was held on 1 September 2022 to practise using the digital platforms. This briefing session also acted as an opportunity for delegates to start networking and to inform delegates about wider BioAirNet aims and work. This session was recorded so those unable to attend could watch in their own time.

Each workshop was focussed on a pre-determined theme. Day 1 was on exploring and conceptualizing, to share, review and build upon current experiences when assessing bioaerosols exposure and their associated health impacts, and what improvements could be made. Day 2 focussed on prioritizing the main challenges that need to be addressed to bridge the gap between current reality and good practice ideals. Day 3 centred on consolidation and action planning to agree ways of working beyond the workshops.

An in-person follow-up workshop was held on 23 November in Birmingham, UK, facilitated by Lucy Brownsdon from the Centre for Facilitation. The workshop was attended by 15 people from the UK and the Netherlands, 10 of whom had attended the original workshops. The purpose of this workshop was to (i) provide update on the projects agreed in the 2022 workshops, (ii) get constructive feedback and input on how to progress the projects further and (iii) continue fostering collaborations.

## Workshop overview and outputs

An overview of the processes used in each workshop is summarized here; for more detail, see Supplementary Material (SM) A.

### Workshop 1: exploring and conceptualizing

The focus of this workshop was on networking, developing a collaborative team and exploring a shared vision for bioaerosols and human health. Three main activities were undertaken in this workshop:

- Exploring a future vision.
- Sharing current practice.
- Identifying future challenges.

Delegates were also asked to share any resources that they found useful (provided in SM B).

#### Exploring a future vision

Delegates were asked for ideas that would lead to the future vision, indicating whether this was directly related to bioaerosol exposure assessment or associated cellular/molecular responses and health impacts, or both. Responses were captured on Mural (SM A). The outputs of this exercise are summarized below:

##### Bioaerosol exposure assessment

- Real-time monitoring and measurement to provide high temporal granularity and better understand emission mechanisms that can then inform dispersion modelling and exposure assessments.
- Speciation of bioaerosol components in real or high-time to improve the characterization of the physiochemical and biological properties of bioaerosols.
- Nationwide surveillance of background bioaerosols to provide exposure estimates commensurate with risk and monitoring drivers, and robust spatially representative baseline data so that changes can be identified (e.g. contributions of anthropogenic sources to background levels, and climate change impacts).
- Improved understanding of how bioaerosols interact with other air pollutants and how they adapt to contaminated environments.

##### Cellular and molecular responses and health impacts

- Physiologically relevant *in vitro* model systems that accurately mimic the complexities of the respiratory system.
- Identification of robust and specific biomarkers of exposure and/or effect that could help to build adverse outcome pathways for improved risk assessment.
- Direct comparison of the effects of individual and mixtures of different bioaerosol components.

##### Both bioaerosol exposure assessment and associated health impacts

- Easily accessible health, demographic and confounding data for use in epidemiological studies to link specific exposures and outcomes.
- Large population scale and longitudinal health cohorts to conduct improved epidemiological studies for understanding how exposures impact health across the human life-course.
- Health-relevant thresholds (e.g. an improved understanding of the dose–response relationship).
- Secure but accessible platforms for sharing data (including exposure, health, demographic and confounder data), knowledge, skills and methods.
- Increased awareness of bioaerosols and their potential health impacts with the public, policymakers and funders.
- Gaps in knowledge, such as the burden of antimicrobial-resistant (bioAMR) bioaerosols and their sources.
- Standardized models/methods (both exposure and biological) to enable interstudy comparison.

### Sharing current practice

Delegates were invited to share current good practices from their fields that would contribute to our shared vision in sub-groups. These included:

- Benefits of interdisciplinary collaborations: working across different sectors and areas of expertise, and with different stakeholders to fill gaps in knowledge using multiple pathway ‘One Health’ approaches.
- Positive experiences of using frameworks to encourage safe sample, data and knowledge sharing, increase communication with each other and between additional stakeholders, and make maximum use of resources.
- Standardization of methods from the project outset to allow comparability between datasets.
- Successful engagement with the public and policymakers, which has increased existing interest and improved awareness of bioaerosols with the relevant people. This has included improved risk communications, offering reassurance and information to moderate concern, and explaining the need for further research in indoor environments.
- Benefits of additional training and funding resources to support collaboration (something that BioAirNet has been able to provide with travel and subsistence funding).

Here, stakeholders are defined as anyone who has an interest in or is affected by bioaerosols. Stakeholders can include, for example, academics, scientists, policymakers working in government, funders or members of the public who suffer with allergies.

### Identifying future challenges

Delegates were asked to identify the gaps between current practice (3.1.2) and our future vision (3.1.1), transferring key answers onto a ‘rocky road’ within Mural (SM A). Key areas were highlighted using ‘hazard signs’.

After workshop 1, but prior to workshop 2, the core team collated the contributions on the rocky road and grouped them into core areas, with priority given to those assigned the most ‘hazard signs’, as follows:

#### Modelling

- Atmospheric modelling (e.g. source and dispersion modelling).
- Cell-based models (i.e. to create relevant *in vitro* airway models and link them to *in vivo* inhalation models and ultimately human cohorts).

#### Measurement of biological responses and health outcomes

- Inflammatory responses to individual and mixtures of bioaerosol components, recognizing that responses may be different between individuals.
- Relevant positive and negative key health endpoints to specific environmental settings or bioaerosol components.
- Standardized methods for systematic and structured comparability.
- Real-time measurement of bioaerosols (including their composition) and health data.

#### Public health impact studies

- Methods to better link epidemiological data with biological models and health outcomes, in multiple directions (so that data from epidemiological studies can be validated or tested in biological models, and molecular processes discovered in biological models can be examined in epidemiological studies).
- Exposure assessment and epidemiological studies to understand health impacts at large population scales, which is important for assessing public health impacts, informing health economics work to determine societal/health care costs associated with bioaerosol exposure and evaluating the effectiveness of interventions.
- Reconciled temporal and spatial scale differences.

#### Data sharing hub

- Secure and easy access (for all) to samples and data (with corresponding metadata) to facilitate multidisciplinary analysis, support future collaboration and share knowledge and skills (including protocols), with appropriate credit and acknowledgement given to sample/data/knowledge generators/owners.
- Opportunities to integrate with existing wider air quality data hubs.
- Defined, clear terminology to promote effective communication between disciplines and enhanced multidisciplinary and collaborative research to address interdisciplinary challenges.

Some other priorities were highlighted but fell outside the scope of the overall aim of this workshop and the human health, behaviour and wellbeing theme of BioAirNet (Theme 3). These included improved bioaerosol measurement, characterization and standardization (covered by BioAirNet Theme 2), engagement (covered by BioAirNet Theme 4) and literature reviews (deemed too resource-intensive to pursue further at this time).

### Workshop 2: prioritizing the main challenges

Workshop 2 focussed on the process of interdisciplinary working and progressing work on the four priority areas identified in workshop 1. The workshop then consisted of three main parts: (i) exploring the four focus areas, (ii) further developing ideas and (iii) discussing how to move forward.

#### Exploring the four focus areas

Delegates joined one of four groups, each representing a core area (modelling, measurement of biological responses and health outcomes, public health impact studies and data sharing hub). Delegates were then asked what they thought the current challenges were; to re-explore the future vision; and to identify what research needs to be conducted, what questions need to be addressed and what insights are needed to achieve the future vision (see SM A for details). Delegates were then brought back together to summarize the key points, which are outlined in Table 1.

**Table 1.** Outcomes when exploring the four focus areas in workshop 2

Core theme: modelling	
**Challenges**	Complete model from molecular through exposure to epidemiological outcome Linking modelling and monitoring Moving from idealized/simplified systems to realistic multipollutant models
**Aspirations**	Source-pathway-exposure outcome model Multipollutant model to capture the exposure and health outcome interactions
**Insights needed**	Academic empathy: understanding different parts of the model pathway A truly interdisciplinary and integrated team Markers and surrogates
**Possible solutions**	Subgroups work on different modules that fit into a conceptual model of source pathway exposure outcome Identify ‘owners’ for sub-models Understand and jointly own the interfaces Collate data that is already available and add bioaerosol data to existing air composition databases Fully understand the composition of air pollution (over time and space) Iterative process to identify the priority combinations to test Carry out cell-level work with single bioaerosols, mixtures and non-biological particles To agree and develop cell models that are widely accepted and commonly used so results are comparable Data to develop/verify models Interaction between modelling and monitoring disciplines to agree and develop what is needed
**Core theme: measurement of biological responses and health outcomes**	
**Challenges**	Techniques not available Speciation; pollen, bacteria, fungi, etc. and viable and non-viable components in both indoor and outdoor environments Teasing out positive vs negative responses Developing methods for specific research questions – need to scale up to wider applications Knowing what you can do, what you can use it for, what you are measuring and what this means Informing risk assessments and guidelines Knowing hazard and exposure to calculate risks
**Aspirations**	Identify/set thresholds relevant for human health Assess species Understand the health responses – potential to categorize the type of response, e.g. whether positive/negative) Set of tests/screens to rank hazards and then prioritize further work – both in terms of research and risk assessment Improved risk assessments Improved links to town planning Ways of boosting exposures to bioaerosols that have positive health impacts
**Insights needed**	Knowledge transfer – the more accessible it is for all, the more you learn – guides development of technical/practical solutions Identifying thresholds for how much you need to be exposed to before a biological response occurs Identifying problem species Continually ask – so what – how does this relate to health and/or regulation? Continued reflection on progress made/what is known to highlight knowledge gaps Link with cellular models to identify those that can be used to measure health impacts
**Possible solutions**	The government passing a law to legislate bioaerosol thresholds to stimulate industry solutions Advanced spectroscopy techniques, machine learning and AI and image recognition techniques Faster and smaller biological assays Biomarkers for health studies Better biological models for testing (e.g. artificial lung) Improved bioaerosol capture and detection techniques (e.g. PM_2.5_ sensor with extra features) Give a Nobel prize to air pollution
**Core theme: public health impact studies**	
**Challenges**	Making sure bioaerosols are not overshadowed by chemicals Knowing what data are available and what data gaps there are Linking different datasets from different specialisms One Health approaches Communication with stakeholders
**Aspirations**	Better baseline data Identifying diversity of organisms/community Understanding sentinels for different environments
**Insights needed**	Identifying parameters needed in particular environmental settings for health assessment Identifying knowledge gaps – literature reviews/desk work prior to field sampling
**Possible solutions**	Help quantify public health and economic burden of bioaerosols Stakeholder mapping Deployment of more online monitoring/sensors to improve baseline data Literature reviews to identify available data and gaps Compiling and sharing data, methods and resources Improved education, student training and citizen engagement – outreach projects, to form part of national curriculum Policy engagement – white papers, etc.
**Core theme: data sharing hub**	
**Challenges**	Who owns the data? General Data Protection Regulation (GDPR) Enormous size of datasets generated by modern machines Insufficient infrastructure for data management Datatypes are driven by type of sampler How do we generate harmonization? Need for skills to develop and maintain such a platform Fear of adversarial science – problems with open access Multiple datatypes, data formats and metadata
**Aspirations**	Harmonization of data for end-point analysis (e.g. health outcomes) Universal standards Open access Gold standard annotation Ability to correct bias
**Insights needed**	Identify/set thresholds relevant for human health Bioaerosol specific data hubs Requires a trusted community to begin with Fair data principles
**Possible solutions**	Identify/set thresholds relevant for human health Allow anonymized release of government datasets Universal metagenomic databases that real-time devices can query UKRI is in a position to lead on requirements – and can indeed enforce them Zenodo-like platforms [22] Best practice guidance for data collection [23] Categorize datasets based on analytical approaches and organize data accordingly Guidelines appropriate to analytical approach Need to know what we have got before we can share it and use it more widely

#### Further developing ideas

The goal of this session was to broaden ideas for the future agenda by encouraging blue-sky thinking, using six creative questions (see SM A for details). Possible solutions identified from this session are captured in Table 1.

#### Discussion on how to move forwards

The aim of this session was to agree an agenda for workshop 3. Delegates could suggest proposals for collaborations. All other participants were encouraged to, first, add ideas to the proposals and, second, show interest in which proposal(s) they wanted to join and progress during workshop 3. This resulted in five final themes:

- A conceptual model for bioaerosol exposure and human health
- A stakeholder mapping exercise
- Drafting a proposal to allow easier knowledge transfer
- A writing project to review emerging opportunities for bioaerosol measurement
- Designing a conference style event for bioaerosols

### Workshop 3: generating outputs and plans for the future

Workshop 3 focussed on further developing the five themes agreed in the previous workshops to generate, and initiate progress on, agreed action plans so momentum could easily continue after the workshops ended. A summary of discussions is provided in Table 2. This enabled actions to be identified and assigned, and timelines to be agreed. The leads of each theme then gave an overview of their action plans to all delegates in a final round-up session. The core team met a week later, on 7 October 2022, to reflect on what was achieved and next steps. The Mural session has remained open, so that all can look back on what was discussed and agreed, which has proved useful for maintaining momentum.

**Table 2.** Summary of action plan development from workshop 3

Theme	Question	Answer
**A conceptual model for bioaerosol exposure and human health**	What is our focus?	To create a conceptual model with sub-models including, but not limited to, molecular effects, dispersion, exposure assessment, health outcomes and clear links co-owned by the sub-models they connect
	What is the output?	A framework showing sources, pathways and exposure impacts that can be used to identify evidence gaps, prioritize research needs and assess risk (with an agreed level of specificity) A model that can feed into Theme 4
	How might we get started?	Can tackle from either end [i.e. risk of outcome (e.g. a disease) or source to assess risk] Sources – what is the source, why is it there and what determinants are there and why? Pathways – means of elucidating pathways, direct exposure (inhalation), indirect exposure [contaminated surfaces (dermal), water and food (ingestion)] Outcome(s) – consider individual and population level, and molecular and pathological pathways from exposure to outcome Start drawing – get contributions for each stage and interconnections
**A stakeholder mapping exercise**	What is our focus?	To map key bioaerosol-related stakeholders, how we engage with them, their level of importance, who we already have links with, how we can engage with them moving forwards to mobilize knowledge and make an impact
	What is the output?	Comprehensive map of key stakeholders and the interactions/relationships between them
	How might we get started?	Bring together work already started as part of BioAirNet Theme 4 and the Environmental Exposures and Health HPRUs at Imperial College London and the University of Leicester Clean up existing spreadsheets into a shareable format Ask BioAirNet members and their wider networks to add to spreadsheet Explore further resources to help drive this forward
**Drafting a proposal to allow easier knowledge transfer**	What is our focus?	To exploit/maximize impact of results, facilitate knowledge transfer through a data hub and identify future collaborative opportunities
	What is the output?	Promote knowledge transfer via seminars and training materials and hands on workshops A white paper on the importance of assessing exposure to bioaerosols (including how they may impact/be impacted by the United Nation’s sustainable development goals, the circular economy and climate change)
	How might we get started?	Use stakeholder map generated by Theme 2 to identify key stakeholders for each target audience (e.g. indoor air, exposure assessments, food safety, antimicrobial resistance, one health interventions, workers and workplace exposure/risk assessors) – so may need to start after Theme 2 completed Prioritize key stakeholders/audiences Identify what knowledge is already available to different stakeholders/audiences Develop a knowledge transfer hub
**A writing project to review emerging opportunities for bioaerosol measurement**	What is our focus?	GAP analysis
	What is the output?	Peer-reviewed paper on how measurement translates to exposure assessment and health impacts
	How might we get started?	Use the Air Quality Expert Group (AQEG) bioaerosol scoping note ‘New opportunities for air pollution measurement’ to develop ideas for a larger ‘Review on emerging opportunities for bioaerosol measurement’ for a peer-reviewed journal Set up a shared/collaborative document Also consider writing up outcomes of these workshops
**Designing a conference style event for bioaerosols**	What is our focus?	To create a regular catch up as a way of maintaining momentum
	What is the output?	Conference/meeting that facilitates networking and collaborative discussions as well as scientific presentations
	How might we get started?	Determine what style of event members would prefer, frequency, remote vs in-person Explore options to host satellite meetings as part of larger existing conferences, promoting bioaerosols wherever possible

## Updates on the action plan and their potential impacts

**Fig. 2.**
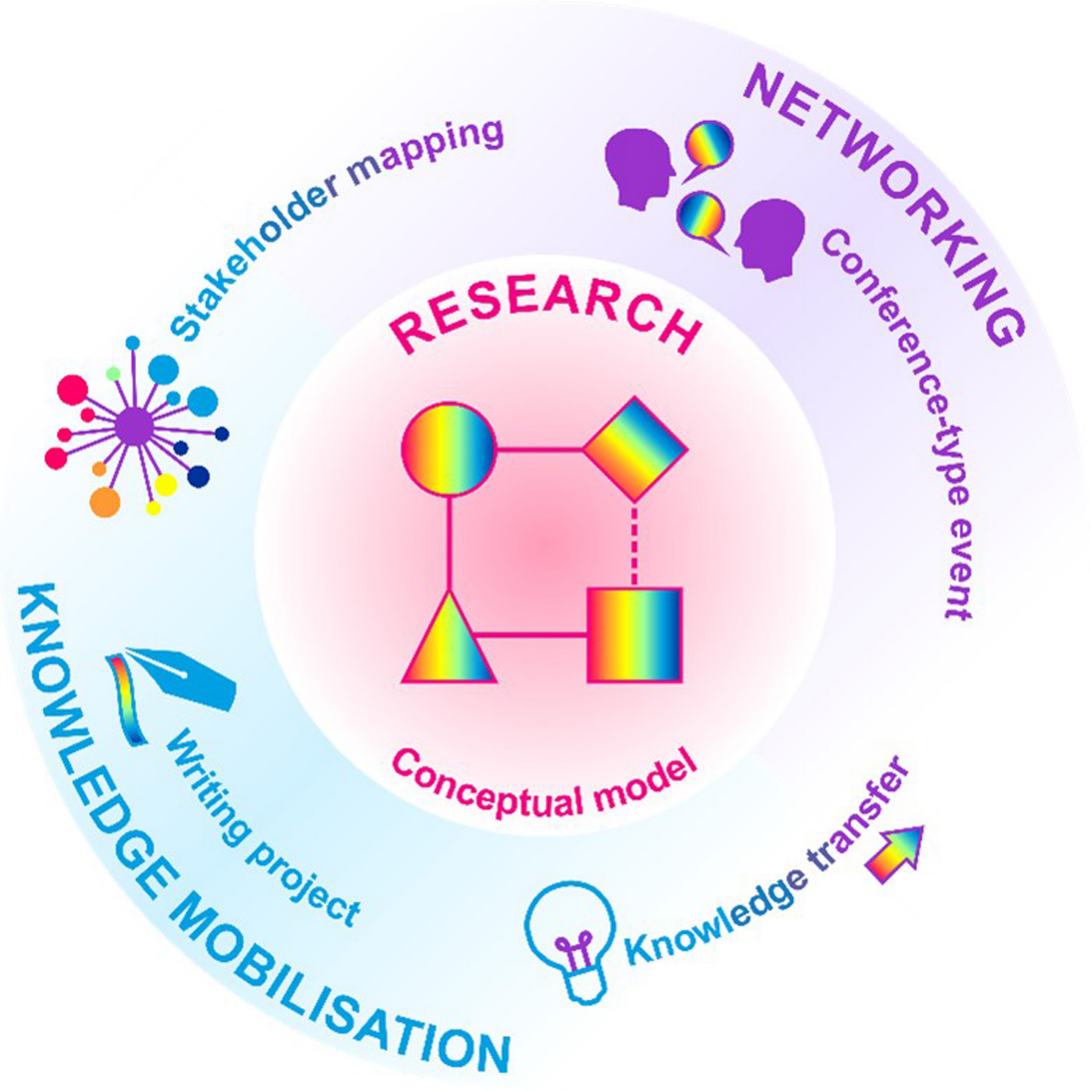
Summary of the five selected themes, the areas they cover and how they interact. The conceptual model is a research tool to better understand and link specific exposure and health outcomes. Research is supported by both knowledge mobilization and networking to ensure it is fit-for-purpose and translated into action. Stakeholder mapping (knowledge transfer and networking) will ensure the right people come together at the right time. Knowledge transfer (knowledge mobilization and networking) will facilitate sharing and collaboration. The writing project (knowledge transfer) will raise awareness and summarize current knowledge for research prioritization. The conference-type event (networking) will maintain momentum by providing an opportunity to share progress and develop new ideas.

In the follow-up workshop, it was clear that progress had been made on the five themes, with some at a more advanced stage than others. Below we summarize the feedback obtained from the 2023 follow-up workshop and the progress made (as of March 2025) on each of the themes. The five themes cover research, knowledge mobilization and networking, as summarized in Fig. 2.

### Completed themes

Three themes are completed within the scope of what was agreed and set out within the realms of the workshops.

#### Stakeholder mapping exercise

Further support was provided by BioAirNet, in the form of a funded 4-month research project, to comprehensively map stakeholders connected to bioaerosols. Expertise in data management and visualization, environmental management and knowledge mobilization was combined to develop a stakeholder map and explore (current and potential future) relationships and key interactions between different stakeholders. This was further discussed and critiqued at two bespoke online workshops on 9 and 16 October 2023. This provided valuable information on stakeholder types/sectors, levels of interest/influence, subject areas, skill types and key funders/regulators/policymakers. A total of 129 stakeholder organizations were identified, mostly from the UK, including representatives from academia, national government, local government, industry, non-profit organizations and networks and societies. At the time of writing, a manuscript summarizing this work has been submitted to a peer-reviewed journal. As a community, we can use this to drive improved skill and data sharing, knowledge mobilization, co-production and multidisciplinary collaborative working, effective communication and strategic foresight, all of which are essential to ensure future research addresses the right questions at the right time, involves and is communicated with the right stakeholders and makes a measurable difference to public health.

#### Knowledge transfer project

A detailed dissemination and communication plan to maximize the effectiveness and impact of communication and dissemination efforts has been drafted. The plan outlines: (1) the aims of communication and dissemination; (2) the specific target audiences, including a complete database of stakeholders (as per the stakeholder mapping exercise described in 4.1.1); and (3) the appropriate channels for reaching the right audiences. Results exploitation should focus on: (a) building contacts with different specialists (exposure assessors) and stakeholders; (b) organizing informative sessions with public authorities, researchers, educational entities and decision makers; and, (c) producing policy briefs to provide a concise, targeted summary of options and recommendations. A draft work package for communicating, disseminating and exploiting results is provided in SM C.

#### Conference-type event

A small survey was sent to the 2022 delegates, after the workshop to explore how people wanted to meet and interact moving forward (SM D), with further discussion on the follow-up workshop held on 23 November. The majority of delegates wanted to meet every 6 months, alternating between shorter online and longer in-person meetings. There was a wish for research presentations, with time incorporated to developing networks and collaborations. It was acknowledged by delegates that there was potential to have bioaerosol-specific forums as part of existing larger conferences and meetings [e.g. the European Geosciences Union (EGU), Aerosol Society, etc.), but existing conferences on specific topics may not be appropriate due to the interdisciplinary nature of bioaerosols. The main barriers included funding and administrative support, although this could potentially be rotated. Since November 2023, a bioaerosol session has been included in the EGU annual conference, with a second planned for 2025. We have also been making use of the extended networks formed during the original workshops to form more specific applied bioaerosol working groups, including establishing regular meetings of a forum for new cutting-edge bioaerosol monitoring instruments.

### Themes that are still in progress

Two themes are still in progress.

#### Conceptual model for bioaerosols and human health

**Fig. 3.**
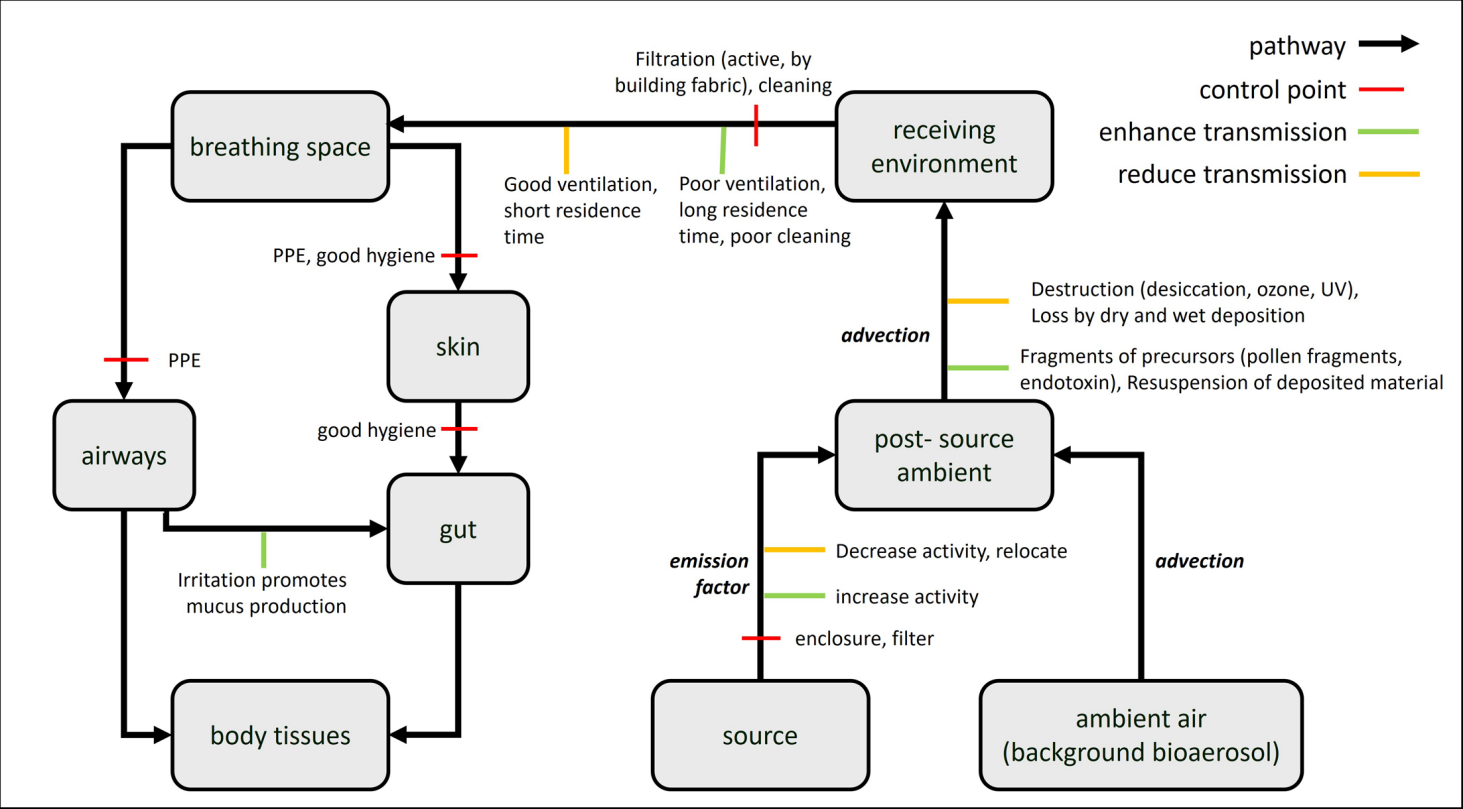
An illustration of the conceptual model, where the grey boxes represent different compartments in which bioaerosols can be found.

A dynamic compartmental model framework was presented, in which the compartments represent spaces (actual or ‘virtual’) where bioaerosols can be found (e.g. headspace above source or breathing zone of individuals). These are interconnected by dynamic processes that drive the accumulation or loss of bioaerosols in a compartment, such as advection or deactivation. The objective of such a model is to determine the relationship between sources and exposure/impacts and to identify control points that might be used to keep the latter at acceptable levels. An illustration of the conceptual model is provided in Fig. 3.

The next step is to collate evidence and knowledge gaps to quantify the dynamic processes.

#### Writing project

There is a perceived need to capture the strengths of contemporary research into bioaerosols that integrates the main public health needs with the rapid pace of development in bioaerosol surveillance using modern methods, both ‘offline’, such as metagenomics, and ‘online’, such as fluorescence-based instruments. A comprehensive summary of sampling methods and analytical techniques to characterize bioaerosols was published as part of another theme within BioAirNet, which provides a ‘toolbox’ of techniques, workflows and technologies for scientists working in the bioaerosols field [1]. However, we still need to better understand where the gaps in surveillance lie, and how technology advances can be democratically used to improve our understanding of human exposures in different settings, be they outdoors, within our homes or in our occupational settings, and the biological mechanisms that link specific exposures with outcomes. To achieve this, a broad range of disciplines and experts are required, and we are exploring these as part of 4.1.1. It is also essential that we are all using the same and/or have the same understanding of key bioaerosol terms. Therefore, since the workshops, BioAirNet has formed a collaborative writing project to explore this common language across different disciplines.

## Conclusion

The BioAirNet initiative, funded by the UKRI SPF, has brought a novel and impactful approach to understanding the complexities of bioaerosol exposure and its implications for human health. By convening multidisciplinary experts through a series of facilitated workshops, BioAirNet has successfully addressed critical gaps in our understanding of bioaerosol diversity and composition. These workshops have not only highlighted current challenges but also provided a platform for collaborative problem-solving and action planning. These efforts are pushing forward the boundaries of knowledge in bioaerosol exposure assessment and shedding light on the molecular and cellular mechanisms influencing health outcomes. By pinpointing critical themes, such as conceptual modelling, stakeholder mapping, knowledge transfer, writing projects, and conference-style events, BioAirNet has laid out a comprehensive roadmap for guiding future research initiatives and fostering collaborative efforts. Further to this, the adoption of online technological advancements and remote working cultures has enabled the effective engagement of a global community, fostering inclusivity and diversity in scientific discussions.

The five themes created here covered research, knowledge mobilization and networking. The conceptual model (research) will build tools to better understand and link exposure measurement through exposure assessment to specific biological responses and health outcomes. Stakeholder mapping (knowledge transfer and networking) will ensure that the right people are involved with the right projects at the right time, maximizing translation of research into actions to improve and protect people’s lives. Knowledge transfer (knowledge mobilization and networking) will facilitate data, samples, knowledge and skill sharing to maximize collaboration and impact. While the five themes identified cover a broad spectrum of the challenges relating to bioaerosol exposure and health outcomes identified during these workshops, they will not address them all. It is hoped that the outcomes of this workshop will act as a toolkit and platform for others to address the remaining key gaps in knowledge identified during this multidisciplinary exercise.

Professional facilitation was key to keeping focus on the topic, ensuring all opinions were heard and considered, and generating an achievable action plan to maintain momentum following the workshops. The success of this initiative highlights the importance of collaborative and multidisciplinary approaches in tackling public health challenges. Therefore, we highly recommend the adoption of similar approaches by scientific communities to foster innovation, inclusivity and impactful partnerships, ultimately leading to tangible improvements in public health and wellbeing.

## Supplementary material

10.1099/mic.0.001561 Uncited Supplementary Material 1.

## Abbreviations

BioAirNet: Indoor/Outdoor Bioaerosols Interface and Relationships Network
BioPM: biological particulate matter
EGU: European Geosciences Union
SPF: strategic priorities fund
UKRI: UK Research and Innovation

## References

[1] Whitby C, Ferguson RMW, Colbeck I, Dumbrell AJ, Nasir ZA, Bohan DA, Dumbrell A (2022). Advances in Ecological Research.

[2] Douglas P, Robertson S, Gay R, Hansell AL, Gant TW (2018). A systematic review of the public health risks of bioaerosols from intensive farming. Int J Hyg Environ Health.

[3] Ghosh B, Lal H, Srivastava A (2015). Review of bioaerosols in indoor environment with special reference to sampling, analysis and control mechanisms. Environ Int.

[4] Robertson S, Douglas P, Jarvis D, Marczylo E (2019). Bioaerosol exposure from composting facilities and health outcomes in workers and in the community: a systematic review update. Int J Hyg Environ Health.

[5] Xie W, Li Y, Bai W, Hou J, Ma T (2021). The source and transport of bioaerosols in the air: a review. Front Environ Sci Eng.

[6] Löndahl J, Möller W, Pagels JH, Kreyling WG, Swietlicki E (2014). Measurement techniques for respiratory tract deposition of airborne nanoparticles: a critical review. J Aerosol Med Pulm Drug Deliv.

[7] Oberdörster G (1993). Lung Dosimetry: Pulmonary Clearance of Inhaled Particles. Aerosol Sci Tech.

[8] Flies EJ, Jones P, Buettel JC, Brook BW (2020). Compromised ecosystem services from urban aerial microbiomes: a review of impacts on human immune function. Front Ecol Evol.

[9] Kirjavainen PV, Karvonen AM, Adams RI, Täubel M, Roponen M (2019). Farm-like indoor microbiota in non-farm homes protects children from asthma development. Nat Med.

[10] Robinson JM, Breed MF (2023). The aerobiome–health axis: a paradigm shift in bioaerosol thinking. Trends Microbiol.

[11] Man WH, de Steenhuijsen Piters WAA, Bogaert D (2017). The microbiota of the respiratory tract: gatekeeper to respiratory health. Nat Rev Microbiol.

[12] Viegas S, Viegas V, Martins C, Assuncao R (2020). Occupational exposure to mycotoxins-different sampling strategies telling a common story regarding occupational studies performed in portugal (2012-2020). Toxins.

[13] BioAirNet Indoor/Outdoor Bioaerosols Interface and Relationships Network [accessed 2024]. https://bioairnet.co.uk/.

[14] Centre for Facilitation What we do [accessed 2024]. https://www.centreforfacilitation.co.uk/.

[15] SESSIONLAB SessionLab [accessed 2024]. https://app.sessionlab.com/signin.

[16] Centre for facilitation (2021). Ground-breaking collaboration in an online world. https://www.centreforfacilitation.co.uk/files/public/BioSkyNet%20Case%20Study%20.pdf.

[17] Liberating Structures Liberating Structures. Including and unleashing everyone [accessed 2024]. https://www.liberatingstructures.com/.

[18] Open Space World (2024). What is Open Space Technology?. https://openspaceworld.org/wp2/what-is/.

[19] Zoom Communications, Inc (2024). Zoom. https://zoom.us/.

[20] Mural [accessed 2024]. https://start.mural.co/free-forever?utm_medium=paid-search&utm_source=adwords&utm_campaign=11265145092&utm_campaign_id=11265145092&utm_term=mural&gad_source=1&gclid=Cj0KCQiAoeGuBhCBARIsAGfKY7xSINbz7AZUI_A6ensgtjRTKDb7m1vN7cpiCxF9M1MuIqs7Ftm3KnYaAvfxEALw_wcB.

[21] Goode EJ, Douglas P, Marczylo E (2022). Understanding the public health implications of bioaerosols. chemical hazards and poisons report.

[22] Zenodo [accessed 2024]. https://zenodo.org/.

[23] Huffman JA, Perring AE, Savage NJ, Clot B, Crouzy B (2019). Real-time sensing of bioaerosols: review and current perspectives. Aerosol Sci Technol.

