# Supplementary material for: Promoting global collaboration to improve bioaerosol exposure assessment and understanding of associated health impacts: outcomes from a series of workshops
Source: Microbiology (Reading). 2025 May 15;171(5):001561. doi: 10.1099/mic.0.001561 (PMC12081852; doi:10.1099/mic.0.001561)
Supplement: Uncited Supplementary Material 1. [file mic-171-01561-s001.pdf]

# Supplementary Material for:

**Promoting global collaboration to improve bioaerosol exposure assessment and understanding of associated health impacts: Outcomes from a series of workshops.**

Emma L Marczylo, Simon Jackson, Christine Bell, Daniel Andrews, Martin J. D. Clift, Ian Crawford, Gyorgy Fejer, Robert M.W. Ferguson, Matthew C. Fisher, Emma-Jane Goode, James Isaac, Rob Kinnersley, Julie A. Morrissey, Sofya Pozdniakova, Carla Viegas, Andrew Ward, Inge M. Wouters, Frederic Coulon, Zaheer A Nasir*, Philippa Douglas

*Corresponding author:

z.a.nasar@cranfield.ac.uk, Cranfield University, School of Water, Energy and Environment, Cranfield, MK43 0AL, UK

## Contents

Supplementary Material A: Details of the processes used in each workshop ........ 2

Workshop 1: Exploring and conceptualising ........ 2

Workshop 2: Prioritising the main challenges ........ 3

Workshop 3: Generating outputs and plans for the future ........ 4

Supplementary Material B: Useful resources shared by delegates ........ 5

Supplementary Material C: Draft work package on communication, dissemination and exploitation of results and guidelines ........ 6

Supplementary Material D: Post-workshop survey sent to delegates ........ 8

References ........ 10

# Supplementary Material A: Details of the processes used in each workshop

## Workshop 1: Exploring and conceptualising

Exploring a future vision

Delegates were briefed to explore a future vision where we would have all the data, tools and expertise to conduct robust assessments of bioaerosol exposure and associated cellular and molecular responses. There was then a two-minute reflective pause so delegates could imagine what their 'future perfect' looked like, considering the following questions:

- What makes your assessment easy to do?
- What makes it possible to easily compare assessments globally?
- How is this distinctively different to what is happening today?

Delegates were randomly split into five groups for this exercise.

An overview to outline the processes involved is provided in Mural here (note, please 'view as a visitor'. You may need to 'zoom out' or navigate and navigate around the Mural page).

Sharing current practice

After mapping out a future vision, delegates were invited to share current good practices from their fields that would contribute to our shared vision. The brief to delegates was to answer the following question 'what is working well already in bioaerosol exposure assessment and/or the measurement of health outcomes?'. Delegates were randomly split into three groups, with a member of the core team acting as a process guardian for each group. The process guardians encouraged each delegate to speak for two minutes to share a good practice story, with a rotating delegate capturing the discussion. Each sub-group identified 2-3 significant good practices to share with the wider group.

Identifying future challenges
Delegates were asked to reflect on the following questions:

- What are the opportunities to make significant changes to our current practise?
- What gaps and challenges do we need to address?
- What improvements could we make to speed up our progress?
- What would really start to make a difference?

Delegates were randomly split into 6 groups and asked to individually capture their answers to the questions using the mural, and then discuss and assess the thoughts as a group. Each group were then invited to transfer the key answers onto a 'rocky road' within mural. The groups were working simultaneously, so the rocky road was populated by each group collectively in real time. Each delegate was then provided with one 'hazard sign' to highlight what they thought was the most important or significant areas that we should focus on as a community, focussing on those that we can influence and that are in our control. Delegates could either choose their own answers, or those put forward by others (either within their group or the other groups). Those answers that had more than one 'hazard sign' were deemed of more importance.

An overview to outline the processes involved is provided in Mural here (note, please 'view as a visitor'. You may need to 'zoom out' or navigate and navigate around the Mural page).

**Workshop 2: Prioritising the main challenges**

After a reminder of progress from Workshop one and a reflective pause and discussion, delegates were briefed on the process the core team had taken to collate the outcomes from the identifying future challenges exercise in workshop one, to ensure transparency in approach and come to a consensus on the final themes to take forwards.

Exploring the four focus areas

Delegates had the option to join one of four groups, each representing a core area (modelling, measurement of biological responses and health outcomes, public health impact studies, data sharing hub), if they had a strong preference (if they didn't, the core team assigned them to a group). Before moving into the breakout groups, the facilitators briefed delegates on what would happen in the breakouts. They advised each group to assign a process guardian who would facilitate a short icebreaker session where delegates had two minutes to share why they thought the topic requires attention. Delegates were then asked what they thought the current challenges are, and these were captured within a 'challenges' area on the mural. The future vision was then re-explored, in the specific context of the core area, with further discussion on how the future vision is better than the current situation. Comments were captured in an 'aspirations' area on the mural. Each group were then encouraged to identify what research needs to be conducted, what questions need to be addressed, and what insights are needed to achieve the future vision. These were also captured on the mural within 'possible solutions' and 'insights' areas. The Mural areas that each group worked on are provided here:

- The mural for the 'modelling' core area can be accessed **here**.
- The mural for the 'measurement of biological responses and health outcomes' core area can be accessed **here.**
- The mural for the 'public health impact studies' core area can be accessed **here.**
- The mural for the 'data sharing hub' core area can be accessed **here.**

Note, when accessing Mural please 'view as a visitor'. You may need to 'zoom out' or navigate and navigate around the Mural page.

Further developing ideas

The facilitators provided a briefing to explain that delegates would again be split into four groups representing the core areas. Each group would have the option to reveal up to six creative questions to expand their creative problem-solving approach and generate more solutions beyond the 'obvious'. Some groups used some or all the questions, whereas others didn't. The questions were:

- What could we do for free to make progress on this?
- What would Beyoncé do? (This was adapted from "What would famous person do" and was included as an unexpected reference point in an academic discussion to generate laughter and from this creativity.)
- What if we did nothing?
- What would someone do if they wanted to prevent this from succeeding?
- What might be possible with limitless budget and political commitment?

- What would be the most surprisingly unexpected thing that we could do?

There were then opportunities for delegates to swap groups and build upon viable solutions. Solutions were captured on the Mural and added to solutions identified when exploring the four focus areas.

Discussion on how to move forwards

Facilitators reminded delegates of the scope and outcomes of the session, and encouraged delegates to consider projects and ideas that would be the most realistic to progress and see impactful outcomes rapidly i.e., 'the low hanging fruit'. The core team reminded delegates of the importance of collaborative multidisciplinary working and reporting back to the entire group. After a reflective pause, there was an open forum so that delegates could put suggest proposals for collaborations, and record this on the mural. Delegates who put proposals forward were assigned as their leads, and all other participants were encouraged to, first, add ideas to the proposals and, second, show interest in which proposal(s) they wanted to join and progress during workshop three.

**Workshop 3: Generating outputs and plans for the future**

Delegates were encouraged to reflect upon the previous workshops, add any new ideas for additional priority themes, and sign up to the themes they wanted to contribute to via the mural board in preparation for workshop three. It was made clear by the facilitators that the agenda for workshop three was open and flexible, and time was provided at the beginning of the workshop to share any updates/insights on, or new ideas for, collaboration before agreeing on the final agenda. Breakout rooms were then used to enable more detailed discussions on the five final themes, which were facilitated by the original proposers. Each theme was asked to answer the following questions:

- What is our focus?
- What is the output?
- How might we get started?
- Who is involved in this?

Delegates were encouraged and given time to move freely between theme rooms to maximise input.

## Supplementary Material B: Useful resources shared by delegates

Prior and during the workshops, delegates were encouraged to use the Mural area to share any useful resources. The resources shared are provided below.

**Peer-reviewed research papers**

(Baliatsas et al., 2020, Banerjee et al., 2021, Basinas et al., 2012, Borlee et al., 2017, Borlee et al., 2018, Danko et al., 2021, de Rooij et al., 2016, de Rooij et al., 2017, de Rooij et al., 2018, de Rooij et al., 2019a, de Rooij et al., 2019b, De Rooij et al., 2019c, Douglas et al., 2017a, Douglas et al., 2017b, Fejer et al., 2013, Gasparrini, 2021, George et al., 2022, Goncalves et al., 2020, Hicks et al., 2020, Horie et al., 2021, Jiang et al., 2018, Klous et al., 2020, Klous et al., 2018, Kucab et al., 2019, Leppänen et al., 2017, Linde et al., 2023, Liu et al., 2011, Luiken et al., 2020, Medina et al., 2015, Oliveira et al., 2022, Post et al., 2019, Roca-Barcelo et al., 2020, Rolph et al., 2018, Salambanga et al., 2022, Simoes et al., 2022, Smit et al., 2008, Smit et al., 2014, Smit and Heederik, 2017, Swanton et al., 2022, van Dijk et al., 2017, Van Gompel et al., 2020a, Van Gompel et al., 2020b, van Kersen et al., 2022, van Kersen et al., 2020, Viegas, 2021, Viegas et al., 2022, Viegas et al., 2021, Viegas et al., 2020, Wielders et al., 2017, Williams et al., 2019, Zomer et al., 2017)

**Links to calls for funding (which have now expired):**

(CIFAR, 2019, UKRI, 2022)

**Links to websites/other resources:**

(UKHSA, 2022)

**Thoughts from delegates:**

- "For funding purposes we should identify the emergent/critical issues that are being addressed for funding: resistance, climate changes, mycotoxins exposure…. or even settings that are hotspots for exposure but with critical importance for circular economy for instance"
- "I've never used these first hand before, but I'm aware that it's possible to share data publicly via open access, peer reviewed platforms, e.g. Nature Scientific Data, Zenodo"

## Supplementary Material C: Draft work package on communication, dissemination and exploitation of results and guidelines

### Communication and dissemination activities

A detailed dissemination and communication plan to maximise the effectiveness and impact of the communication and dissemination efforts will be performed. The plan will discriminate: 1) the communication and dissemination aims; 2) the specific target audiences including a complete database of stakeholders; and 3) the appropriate channels for the right audience and the specific key message in the right project timeframe.

A communication package to promote the project will be prepared. The project website will be a key tool for communicating the project's relevance and results to a wide audience, but it will also include a member-only section for internal communication. Platforms such as Twitter, YouTube, and Instagram will help share the material with a wider non-specialised audience. In addition, the official European Commission channels and institutional channels of the partners to disseminate the main results will be used. Press releases and project newsletters for national and international mass media when relevant results become available and justify a wider reach will be prepared. Dissemination for the scientific community will mainly be through presentations at scientific events and open access publications in peer-reviewed journals.

### Organisation of webinars, and a conference

An annual webinar/seminar for the dissemination of results and guidelines will be held. The seminars will present our results and will target mainly other researchers, exposure assessors working on occupational assessments, IAQ experts, NGOs, and companies. Summer schools will be held targeting junior researchers in the field contributing to the harmonization of sampling methods and assays employed in the bioaerosols exposure assessment. An International Conference for dissemination of the results, open to all target groups, will be held.

### FAIR datasets preparation and sharing

The project will produce a large amount of data, which will be shared following the FAIR data principles. The datasets will be shared primarily on Zenodo under a Creative Commons license chosen by the owner of the results (Consortium coordination). The deposited data will be supplied with the required standard metadata and persistent identifiers (e.g., DOI, URN). The datasets will be properly anonymised to permit their release and comply with Ethical rules.

### Results exploitation

An exploitation plan will be prepared. Part of this task will focus on building contacts with different specialists (exposure assessors) and stakeholders to ensure long-lasting networks and receive some feedback on the project activities. Informative sessions with public authorities, researchers, educational entities, and decision-makers to raise awareness on the network, our impact and the potential exploitation of our results will be organised. Some of these consultations will target fostering collaboration with other EU projects.

Policy briefs will be prepared to provide a concise, targeted summary of policy options and recommendations, focusing mainly on bioaerosols exposure in different environments. Guidelines and training materials for interventions in the different studied environments will be widely shared for health promotion and disease prevention.

## Supplementary Material D: Post-workshop survey sent to delegates

The following questions and options were sent delegates after the workshops held in September 2022:

1. How frequently would you like to catch up? (Options: Every 6 months, Every year, Other – delegates could choose one option)
2. How would you like to meet? (Options: Virtually, In person, Alternating between virtually and in-person - delegates could choose one option)
3. What would you like meetings to cover? (Options: Specific research topics, Specific funding calls, Recent/new research finding(s), Challenges to address, Collaborative working, Data/sample sharing, Specific conferences, All/Mixture of the above (full day meeting), All/Mixture of the above (half day meeting), All/Mixture of the above (shorter meeting), Other- delegates could choose multiple options)
4. Are there any existing meeting/networks we could piggy back onto to both grow our membership and prevent duplication of efforts? (Free text - - delegates could contribute multiple times)

Fourteen delegates responded. The results are summarised in Figure SM1.

As the majority of delegates wanted to alternate between in-person and virtual meetings, it was decided to hold an in-person workshop in November 2023 as the workshops in September 2022 were virtual.

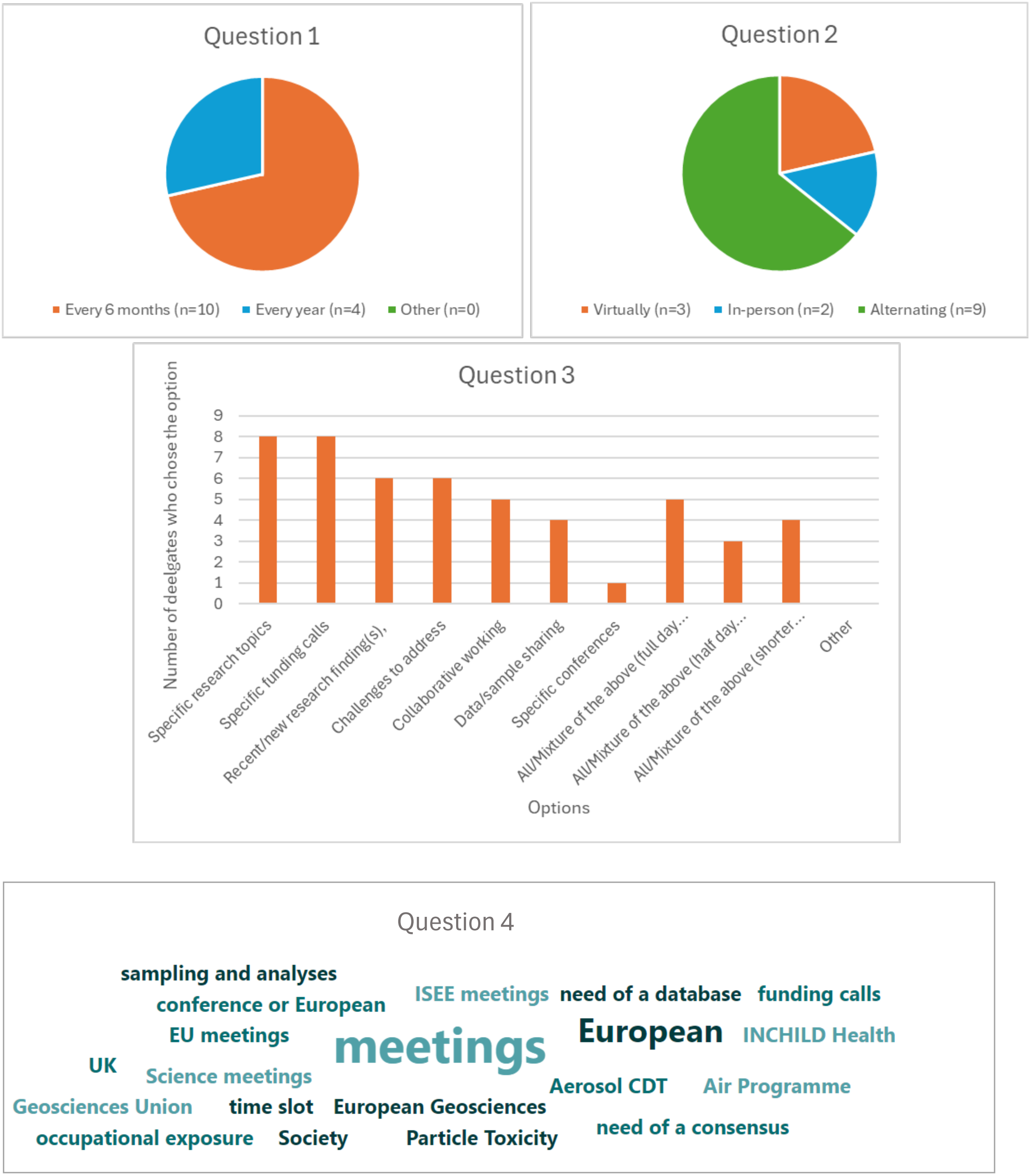


**Figure SM1.** The results of the post-workshop survey sent to delegates.

## References

BALIATSAS, C., DUCKERS, M., SMIT, L., HEEDERIK, D. & YZERMANS, J. 2020. Morbidity Rates in an Area with High Livestock Density: A Registry-Based Study Including Different Groups of Patients with Respiratory Health Problems. *Int J Environ Res Public Health,* 17.

BANERJEE, S., DENNING, D. W. & CHAKRABARTI, A. 2021. One Health aspects & priority roadmap for fungal diseases : A mini-review. *Indian J Med Res,* 153**,** 311-319.

BASINAS, I., SCHLUNSSEN, V., HEEDERIK, D., SIGSGAARD, T., SMIT, L. A., SAMADI, S., OMLAND, O., HJORT, C., MADSEN, A. M., SKOV, S. & WOUTERS, I. M. 2012. Sensitisation to common allergens and respiratory symptoms in endotoxin exposed workers: a pooled analysis. *Occup Environ Med,* 69**,** 99-106.

BORLEE, F., YZERMANS, C. J., AALDERS, B., ROOIJACKERS, J., KROP, E., MAASSEN, C. B. M., SCHELLEVIS, F., BRUNEKREEF, B., HEEDERIK, D. & SMIT, L. A. M. 2017. Air Pollution from Livestock Farms Is Associated with Airway Obstruction in Neighboring Residents. *Am J Respir Crit Care Med,* 196**,** 1152-1161.

BORLEE, F., YZERMANS, C. J., KROP, E. J. M., MAASSEN, C. B. M., SCHELLEVIS, F. G., HEEDERIK, D. J. J. & SMIT, L. A. M. 2018. Residential proximity to livestock farms is associated with a lower prevalence of atopy. *Occup Environ Med,* 75**,** 453-460.

CIFAR. 2019. *Fungal Kingdom: Threats & Opportunities* [Online]. Available: https://cifar.ca/research-programs/fungal-kingdom/ [Accessed].

DANKO, D., BEZDAN, D., AFSHIN, E. E., AHSANUDDIN, S., BHATTACHARYA, C., BUTLER, D. J., CHNG, K. R., DONNELLAN, D., HECHT, J., JACKSON, K., KUCHIN, K., KARASIKOV, M., LYONS, A., MAK, L., MELESHKO, D., MUSTAFA, H., MUTAI, B., NECHES, R. Y., NG, A., NIKOLAYEVA, O., NIKOLAYEVA, T., PNG, E., RYON, K. A., SANCHEZ, J. L., SHAABAN, H., SIERRA, M. A., THOMAS, D., YOUNG, B., ABUDAYYEH, O. O., ALICEA, J., BHATTACHARYYA, M., BLEKHMAN, R., CASTRO-NALLAR, E., CANAS, A. M., CHATZIEFTHIMIOU, A. D., CRAWFORD, R. W., DE FILIPPIS, F., DENG, Y., DESNUES, C., DIAS-NETO, E., DYBWAD, M., ELHAIK, E., ERCOLINI, D., FROLOVA, A., GANKIN, D., GOOTENBERG, J. S., GRAF, A. B., GREEN, D. C., HAJIRASOULIHA, I., HASTINGS, J. J. A., HERNANDEZ, M., IRAOLA, G., JANG, S., KAHLES, A., KELLY, F. J., KNIGHTS, K., KYRPIDES, N. C., LABAJ, P. P., LEE, P. K. H., LEUNG, M. H. Y., LJUNGDAHL, P. O., MASON-BUCK, G., MCGRATH, K., MEYDAN, C., MONGODIN, E. F., MORAES, M. O., NAGARAJAN, N., NIETO-CABALLERO, M., NOUSHMEHR, H., OLIVEIRA, M., OSSOWSKI, S., OSUOLALE, O. O., OZCAN, O., PAEZ-ESPINO, D., RASCOVAN, N., RICHARD, H., RATSCH, G., SCHRIML, L. M., SEMMLER, T., SEZERMAN, O. U., SHI, L., SHI, T., SIAM, R., SONG, L. H., SUZUKI, H., COURT, D. S., TIGHE, S. W., TONG, X., UDEKWU, K. I., UGALDE, J. A., VALENTINE, B., VASSILEV, D. I., VAYNDORF, E. M., VELAVAN, T. P., WU, J., ZAMBRANO, M. M., ZHU, J., ZHU, S., MASON, C. E. & INTERNATIONAL META, S. U. B. C. 2021. A global metagenomic map of urban microbiomes and antimicrobial resistance. *Cell,* 184**,** 3376-3393 e17.

DE ROOIJ, M. M., BORLEE, F., SMIT, L. A., DE BRUIN, A., JANSE, I., HEEDERIK, D. J. & WOUTERS, I. M. 2016. Detection of Coxiella burnetii in Ambient Air after a Large Q Fever Outbreak. *PLoS One,* 11**,** e0151281.

DE ROOIJ, M. M., HEEDERIK, D. J., BORLEE, F., HOEK, G. & WOUTERS, I. M. 2017. Spatial and temporal variation in endotoxin and PM10 concentrations in ambient air in a livestock dense area. *Environ Res,* 153**,** 161-170.

DE ROOIJ, M. M. T., HEEDERIK, D. J. J., VAN NUNEN, E., VAN SCHOTHORST, I. J., MAASSEN, C. B. M., HOEK, G. & WOUTERS, I. M. 2018. Spatial Variation of Endotoxin Concentrations Measured in Ambient

PM(10) in a Livestock-Dense Area: Implementation of a Land-Use Regression Approach. *Environ Health Perspect,* 126**,** 017003.
DE ROOIJ, M. M. T., HOEK, G., SCHMITT, H., JANSE, I., SWART, A., MAASSEN, C. B. M., SCHALK, M., HEEDERIK, D. J. J. & WOUTERS, I. M. 2019a. Insights into Livestock-Related Microbial Concentrations in Air at Residential Level in a Livestock Dense Area. *Environ Sci Technol,* 53**,** 7746-7758.
DE ROOIJ, M. M. T., SMIT, L. A. M., ERBRINK, H. J., HAGENAARS, T. J., HOEK, G., OGINK, N. W. M., WINKEL, A., HEEDERIK, D. J. J. & WOUTERS, I. M. 2019b. Endotoxin and particulate matter emitted by livestock farms and respiratory health effects in neighboring residents. *Environ Int,* 132**,** 105009.
DE ROOIJ, M. M. T., VAN LEUKEN, J. P. G., SWART, A., KRETZSCHMAR, M. E. E., NIELEN, M., DE KOEIJER, A. A., JANSE, I., WOUTERS, I. M. & HEEDERIK, D. J. J. 2019c. A systematic knowledge synthesis on the spatial dimensions of Q fever epidemics. *Zoonoses Public Health,* 66**,** 14-25.
DOUGLAS, P., HAYES, E. T., WILLIAMS, W. B., TYRREL, S. F., KINNERSLEY, R. P., WALSH, K., O'DRISCOLL, M., LONGHURST, P. J., POLLARD, S. J. T. & DREW, G. H. 2017a. Use of dispersion modelling for Environmental Impact Assessment of biological air pollution from composting: Progress, problems and prospects. *Waste Manag,* 70**,** 22-29.
DOUGLAS, P., TYRREL, S. F., KINNERSLEY, R. P., WHELAN, M., LONGHURST, P. J., HANSELL, A. L., WALSH, K., POLLARD, S. J. & DREW, G. H. 2017b. Predicting Aspergillus fumigatus exposure from composting facilities using a dispersion model: A conditional calibration and validation. *Int J Hyg Environ Health,* 220**,** 17-28.
FEJER, G., WEGNER, M. D., GYORY, I., COHEN, I., ENGELHARD, P., VORONOV, E., MANKE, T., RUZSICS, Z., DOLKEN, L., PRAZERES DA COSTA, O., BRANZK, N., HUBER, M., PRASSE, A., SCHNEIDER, R., APTE, R. N., GALANOS, C. & FREUDENBERG, M. A. 2013. Nontransformed, GM-CSF-dependent macrophage lines are a unique model to study tissue macrophage functions. *Proc Natl Acad Sci U S A,* 110**,** E2191-8.
GASPARRINI, A. 2021. The Case Time Series Design. *Epidemiology,* 32**,** 829-837.
GEORGE, P. B. L., ROSSI, F., ST-GERMAIN, M. W., AMATO, P., BADARD, T., BERGERON, M. G., BOISSINOT, M., CHARETTE, S. J., COLEMAN, B. L., CORBEIL, J., CULLEY, A. I., GAUCHER, M. L., GIRARD, M., GODBOUT, S., KIRYCHUK, S. P., MARETTE, A., MCGEER, A., O'SHAUGHNESSY, P. T., PARMLEY, E. J., SIMARD, S., REID-SMITH, R. J., TOPP, E., TRUDEL, L., YAO, M., BRASSARD, P., DELORT, A. M., LARIOS, A. D., LETOURNEAU, V., PAQUET, V. E., PEDNEAU, M. H., PIC, E., THOMPSON, B., VEILLETTE, M., THALER, M., SCAPINO, I., LEBEUF, M., BAGHDADI, M., CASTILLO TORO, A., CAYOUETTE, A. B., DUBOIS, M. J., DUROCHER, A. F., GIRARD, S. B., DIAZ, A. K. C., KHALLOUFI, A., LECLERC, S., LEMIEUX, J., MALDONADO, M. P., PILON, G., MURPHY, C. P., NOTLING, C. A., OFORI-DARKO, D., PROVENCHER, J., RICHER-FORTIN, A., TURGEON, N. & DUCHAINE, C. 2022. Antimicrobial Resistance in the Environment: Towards Elucidating the Roles of Bioaerosols in Transmission and Detection of Antibacterial Resistance Genes. *Antibiotics (Basel),* 11.
GONCALVES, P., MELO, A., DIAS, M., ALMEIDA, B., CAETANO, L. A., VERISSIMO, C., VIEGAS, C. & SABINO, R. 2020. Azole-Resistant Aspergillus fumigatus Harboring the TR(34)/L98H Mutation: First Report in Portugal in Environmental Samples. *Microorganisms,* 9.
HICKS, J. M., SILMAN, N. J., JACKSON, S. K., AYLOTT, J. W. & RAWSON, F. J. 2020. Mass transport of lipopolysaccharide induced H(2)O(2) detected by an intracellular carbon nanoelectrode sensor. *Bioelectrochemistry,* 135**,** 107547.
HORIE, M., YANG, D., JOOSTEN, P., MUNK, P., WADEPOHL, K., CHAUVIN, C., MOYANO, G., SKARZYNSKA, M., DEWULF, J., AARESTRUP, F. M., BLAHA, T., SANDERS, P., GONZALEZ-ZORN, B., WASYL, D., WAGENAAR, J. A., HEEDERIK, D., MEVIUS, D., SCHMITT, H., SMIT, L. A. M. & VAN GOMPEL, L. 2021. Risk Factors for Antimicrobial Resistance in Turkey Farms: A Cross-Sectional Study in Three European Countries. *Antibiotics (Basel),* 10.

JIANG, C., WANG, X., LI, X., INLORA, J., WANG, T., LIU, Q. & SNYDER, M. 2018. Dynamic Human Environmental Exposome Revealed by Longitudinal Personal Monitoring. *Cell,* 175**,** 277-291 e31.

KLOUS, G., SMIT, L. A., VAN DER HOEK, W., KRETZSCHMAR, M. E., VELLEMA, P., COUTINHO, R. A., HEEDERIK, D. J. & HUSS, A. 2020. Relationship between Coxiella burnetii (Q fever) antibody serology and time spent outdoors. *J Infect,* 81**,** 90-97.

KLOUS, G., SMIT, L. A. M., FREIDL, G. S., BORLEE, F., VAN DER HOEK, W., CJ, I. J., KRETZSCHMAR, M. E. E., HEEDERIK, D. J. J., COUTINHO, R. A. & HUSS, A. 2018. Pneumonia risk of people living close to goat and poultry farms - Taking GPS derived mobility patterns into account. *Environ Int,* 115**,** 150-160.

KUCAB, J. E., ZOU, X., MORGANELLA, S., JOEL, M., NANDA, A. S., NAGY, E., GOMEZ, C., DEGASPERI, A., HARRIS, R., JACKSON, S. P., ARLT, V. M., PHILLIPS, D. H. & NIK-ZAINAL, S. 2019. A Compendium of Mutational Signatures of Environmental Agents. *Cell,* 177**,** 821-836 e16.

LEPPÄNEN, H. K., TÄUBEL, M., JAYAPRAKASH, B., VEPSÄLÄINEN, A., PASANEN, P. & HYVÄRINEN, A. 2017. Quantitative assessment of microbes from samples of indoor air and dust. *Journal of Exposure Science & Environmental Epidemiology,* 28**,** 231-241.

LINDE, K. J., WOUTERS, I. M., KLUYTMANS, J., KLUYTMANS-VAN DEN BERGH, M. F. Q., PAS, S. D., GEURTSVANKESSEL, C. H., KOOPMANS, M. P. G., MEIER, M., MEIJER, P., RABEN, C. R., SPITHOVEN, J., TERSTEEG-ZIJDERVELD, M. H. G., HEEDERIK, D. J. J., DOHMEN, W. & CONSORTIUM, C. 2023. Detection of SARS-CoV-2 in Air and on Surfaces in Rooms of Infected Nursing Home Residents. *Ann Work Expo Health,* 67**,** 129-140.

LIU, J., PANKHURST, L. J., DEACON, L. J., ABATE, W., HAYES, E. T., DREW, G. H., LONGHURST, P. J., POLLARD, S., LONGHURST, J., TYRREL, S. F. & JACKSON, S. K. 2011. Evaluation of inflammatory effects of airborne endotoxin emitted from composting sources. *Environ Toxicol Chem,* 30**,** 602-6.

LUIKEN, R. E. C., VAN GOMPEL, L., BOSSERS, A., MUNK, P., JOOSTEN, P., HANSEN, R. B., KNUDSEN, B. E., GARCIA-COBOS, S., DEWULF, J., AARESTRUP, F. M., WAGENAAR, J. A., SMIT, L. A. M., MEVIUS, D. J., HEEDERIK, D. J. J., SCHMITT, H. & GROUP, E. 2020. Farm dust resistomes and bacterial microbiomes in European poultry and pig farms. *Environ Int,* 143**,** 105971.

MEDINA, Á., RODRÍGUEZ, A. & MAGAN, N. 2015. Climate change and mycotoxigenic fungi: impacts on mycotoxin production. *Current Opinion in Food Science,* 5**,** 99-104.

OLIVEIRA, K., VIEGAS, C. & RIBEIRO, E. 2022. MRSA Colonization in Workers from Different Occupational Environments—A One Health Approach Perspective. *Atmosphere,* 13.

POST, P. M., HOGERWERF, L., HUSS, A., PETIE, R., BOENDER, G. J., BALIATSAS, C., LEBRET, E., HEEDERIK, D., HAGENAARS, T. J., CJ, I. J. & SMIT, L. A. M. 2019. Risk of pneumonia among residents living near goat and poultry farms during 2014-2016. *PLoS One,* 14**,** e0223601.

ROCA-BARCELO, A., DOUGLAS, P., FECHT, D., STERRANTINO, A. F., WILLIAMS, B., BLANGIARDO, M., GULLIVER, J., HAYES, E. T. & HANSELL, A. L. 2020. Risk of respiratory hospital admission associated with modelled concentrations of Aspergillus fumigatus from composting facilities in England. *Environ Res,* 183**,** 108949.

ROLPH, C., GWYTHER, C., TYRREL, S., NASIR, Z., DREW, G., JACKSON, S., KHERA, S., HAYES, E., WILLIAMS, B., BENNETT, A., COLLINS, S., WALSH, K., KINNERSLEY, R. & GLADDING, T. 2018. Sources of Airborne Endotoxins in Ambient Air and Exposure of Nearby Communities—A Review. *Atmosphere,* 9.

SALAMBANGA, F. R. D., WINGERT, L., VALOIS, I., LACOMBE, N., GOUIN, F., TREPANIER, J., DEBIA, M., SOSZCZYNSKA, E., TWARUZEK, M., KOSICKI, R., DIAS, M., VIEGAS, S., CAETANO, L., VIEGAS, C. & MARCHAND, G. 2022. Microbial contamination and metabolite exposure assessment during waste and recyclable material collection. *Environ Res,* 212**,** 113597.

SIMOES, M., JANSSEN, N., HEEDERIK, D. J. J., SMIT, L. A. M., VERMEULEN, R. & HUSS, A. 2022. Residential proximity to livestock animals and mortality from respiratory diseases in The Netherlands: A prospective census-based cohort study. *Environ Int,* 161**,** 107140.

SMIT, L. A., HEEDERIK, D., DOEKES, G., BLOM, C., VAN ZWEDEN, I. & WOUTERS, I. M. 2008. Exposure-response analysis of allergy and respiratory symptoms in endotoxin-exposed adults. *Eur Respir J,* 31**,** 1241-8.

SMIT, L. A., HOOIVELD, M., VAN DER SMAN-DE BEER, F., OPSTAL-VAN WINDEN, A. W., BEEKHUIZEN, J., WOUTERS, I. M., YZERMANS, C. J. & HEEDERIK, D. 2014. Air pollution from livestock farms, and asthma, allergic rhinitis and COPD among neighbouring residents. *Occup Environ Med,* 71**,** 134-40.

SMIT, L. A. M. & HEEDERIK, D. 2017. Impacts of Intensive Livestock Production on Human Health in Densely Populated Regions. *Geohealth,* 1**,** 272-277.

SWANTON, C., HILL, W., LIM, E., LEE, C., WEEDEN, C. E., AUGUSTINE, M., CHEN, K., KUAN, F. C., MARONGIU, F., RODRIGUES, F., CHA, H., JACKS, T., LUCHTENBORG, M., MALANCHI, I., DOWNWARD, J., CARLSTEN, C., HACKSHAW, A., LITCHFIELD, K. R., DEGREGORI, J. & JAMAL-HANJANI, M. 2022. LBA1 Mechanism of action and an actionable inflammatory axis for air pollution induced non-small cell lung cancer: Towards molecular cancer prevention. *Annals of Oncology,* 33.

UKHSA. 2022. *UK Health Security Agency. Air quality work in the HPRU Development Award in Environmental Exposures & Health for #CleanAirDay.* [Online]. Available: https://www.youtube.com/watch?v=W_AAO9mUm3Y [Accessed].

UKRI. 2022. *UK Research and Innovation. Partner with researchers in Canada on antimicrobial resistance (AMR)* [Online]. Available: https://www.ukri.org/opportunity/partner-with-researchers-in-canada-on-antimicrobial-resistance-amr/ [Accessed].

VAN DIJK, C. E., ZOCK, J. P., BALIATSAS, C., SMIT, L. A. M., BORLEE, F., SPREEUWENBERG, P., HEEDERIK, D. & YZERMANS, C. J. 2017. Health conditions in rural areas with high livestock density: Analysis of seven consecutive years. *Environ Pollut,* 222**,** 374-382.

VAN GOMPEL, L., DOHMEN, W., LUIKEN, R. E. C., BOUWKNEGT, M., HERES, L., VAN HEIJNSBERGEN, E., JONGERIUS-GORTEMAKER, B. G. M., SCHERPENISSE, P., GREVE, G. D., TERSTEEG-ZIJDERVELD, M. H. G., WADEPOHL, K., RIBEIRO DUARTE, A. S., MUNOZ-GOMEZ, V., FISCHER, J., SKARZYNSKA, M., WASYL, D., WAGENAAR, J. A., URLINGS, B. A. P., DORADO-GARCIA, A., WOUTERS, I. M., HEEDERIK, D. J. J., SCHMITT, H. & SMIT, L. A. M. 2020a. Occupational Exposure and Carriage of Antimicrobial Resistance Genes (tetW, ermB) in Pig Slaughterhouse Workers. *Ann Work Expo Health,* 64**,** 125-137.

VAN GOMPEL, L., LUIKEN, R. E. C., HANSEN, R. B., MUNK, P., BOUWKNEGT, M., HERES, L., GREVE, G. D., SCHERPENISSE, P., JONGERIUS-GORTEMAKER, B. G. M., TERSTEEG-ZIJDERVELD, M. H. G., GARCIA-COBOS, S., DOHMEN, W., DORADO-GARCIA, A., WAGENAAR, J. A., URLINGS, B. A. P., AARESTRUP, F. M., MEVIUS, D. J., HEEDERIK, D. J. J., SCHMITT, H., BOSSERS, A. & SMIT, L. A. M. 2020b. Description and determinants of the faecal resistome and microbiome of farmers and slaughterhouse workers: A metagenome-wide cross-sectional study. *Environ Int,* 143**,** 105939.

VAN KERSEN, W., BOSSERS, A., DE STEENHUIJSEN PITERS, W. A. A., DE ROOIJ, M. M. T., BONTEN, M., FLUIT, A. C., HEEDERIK, D., PAGANELLI, F. L., ROGERS, M., VIVEEN, M., BOGAERT, D., LEAVIS, H. L. & SMIT, L. A. M. 2022. Air pollution from livestock farms and the oropharyngeal microbiome of COPD patients and controls. *Environ Int,* 169**,** 107497.

VAN KERSEN, W., OLDENWENING, M., AALDERS, B., BLOEMSMA, L. D., BORLEE, F., HEEDERIK, D. & SMIT, L. A. M. 2020. Acute respiratory effects of livestock-related air pollution in a panel of COPD patients. *Environ Int,* 136**,** 105426.

VIEGAS, C. 2021. Climate Change influence in fungi. *European Journal of Public Health,* 31**,** iii102.

VIEGAS, C., ALMEIDA, B., ARANHA CAETANO, L., AFANOU, A., STRAUMFORS, A., VERISSIMO, C., GONCALVES, P. & SABINO, R. 2022. Algorithm to assess the presence of Aspergillus fumigatus resistant strains: The case of Norwegian sawmills. *Int J Environ Health Res,* 32**,** 963-971.

VIEGAS, C., CAETANO, L. A. & VIEGAS, S. 2021. Occupational exposure to Aspergillus section Fumigati: Tackling the knowledge gap in Portugal. *Environ Res,* 194**,** 110674.

VIEGAS, S., ASSUNCAO, R., TWARUZEK, M., KOSICKI, R., GRAJEWSKI, J. & VIEGAS, C. 2020. Mycotoxins feed contamination in a dairy farm - potential implications for milk contamination and workers' exposure in a One Health approach. *J Sci Food Agric,* 100**,** 1118-1123.

WIELDERS, C. C. H., VAN HOEK, A., HENGEVELD, P. D., VEENMAN, C., DIERIKX, C. M., ZOMER, T. P., SMIT, L. A. M., VAN DER HOEK, W., HEEDERIK, D. J., DE GREEFF, S. C., MAASSEN, C. B. M. & VAN DUIJKEREN, E. 2017. Extended-spectrum beta-lactamase- and pAmpC-producing Enterobacteriaceae among the general population in a livestock-dense area. *Clin Microbiol Infect,* 23**,** 120 e1-120 e8.

WILLIAMS, B., DOUGLAS, P., ROCA BARCELO, A., HANSELL, A. L. & HAYES, E. 2019. Estimating Aspergillus fumigatus exposure from outdoor composting activities in England between 2005 and 14. *Waste Manag,* 84**,** 235-244.

ZOMER, T. P., E, V. A. N. D., WIELDERS, C. C. H., VEENMAN, C., HENGEVELD, P., W, V. D. H., SC, D. E. G., SMIT, L. A. M., HEEDERIK, D. J., YZERMANS, C. J., KUIJPER, E. J. & MAASSEN, C. B. M. 2017. Prevalence and risk factors for colonization of Clostridium difficile among adults living near livestock farms in the Netherlands. *Epidemiol Infect,* 145**,** 2745-2749.
